# Presence of Human Papilloma Virus in a Series of Breast Carcinoma from Argentina

**DOI:** 10.1371/journal.pone.0061613

**Published:** 2013-04-25

**Authors:** Ana Laura Pereira Suarez, Mario Alejandro Lorenzetti, Rene Gonzalez Lucano, Melina Cohen, Hugo Gass, Paula Martinez Vazquez, Pedro Gonzalez, Maria V. Preciado, Paola Chabay

**Affiliations:** 1 Department of Physiology, University Center for Health Sciences, University of Guadalajara, Guadalajara, Jalisco, Mexico; 2 Molecular Biology Laboratory, Pathology Division, Ricardo Gutiérrez Children Hospital, Ciudad de Buenos Aires, Argentina; 3 Ginecology Division, Magdalena Villegas de Martínez Hospital, General Pacheco, Tigre, Provincia de Buenos Aires, Argentina; 4 Pathology Division, Magdalena Villegas de Martínez Hospital, General Pacheco, Tigre Provincia de Buenos Aires, Argentina; IPO, Inst Port Oncology, Portugal

## Abstract

**Background:**

The etiology and the molecular mechanisms related to breast carcinogenesis remain poorly understood. Some recent reports have examined the role of *Human Papillomavirus* (HPV) in this disease. The purpose of this study was to determine the prevalence of HPV in breast cancer.

**Methods:**

Sixty one fresh frozen breast cancers samples were analyzed. Samples were tested for HPV by PCR, and products were automatically sequenced. Findings were correlated with clinical and pathological characteristics.

**Results:**

The HPV DNA prevalence in the breast cancer samples was 26% (16/61). Clinical parameters were not statistically associated with HPV presence (p>0.05 χ^2^ test). Sequence analysis in a subgroup of cases indicates the prevalence of low risk HPV11, followed by high risk HPV16. We found no HPV transcriptional activity.

**Conclusion:**

The present study demonstrated for the first time in Argentina the presence of HPV in a proportion of the malignant breast tissues. This finding suggests that HPV may have a biological significance in breast carcinogenesis.

## Introduction

Breast cancer is the most frequently diagnosed malignancy in women in many populations [1], and the incidence of breast carcinoma has increased by 2.5% over the last 50 years [2]. Many risk factors have been associated with the pathogenesis of this disease; including family history, hormones, cigarette smoking and alcohol consumption [3], but the molecular mechanisms related to the breast carcinogenesis remain poorly understood. In Argentina, deaths caused by breast carcinoma raise up to 21.8 per every 100,000 women, the second place in Latin America, and represent 5,400 deaths per year [4].

Currently a large number of infectious agents have been described to either cause or contribute to specific human cancers [5]. Indeed, 10–15% of the cancers worldwide can be linked to viral infections [6]. Many studies have recently suggested that certain viruses might be involved in the pathogenesis of breast cancer, such as specific types of Human papillomavirus (HPV), Epstein Barr virus (EBV) and Mouse mammary tumor virus (MMTV), but viral involvement in breast carcinogenesis still remains controversial. While some groups have suggested that high risk HPV could be implicated in breast tumor development [3], [7]–[22], other groups detracted this proposal [23]–[26]. However, HPV immortalization of normal breast epithelium has been demonstrated *in vitro* [27], raising the possibility that HPV might be etiologically related to a proportion of breast cancers.

Human papillomaviruses (HPVs) species, family Papillomaviridae, genus Alphapapillomavirus, are small DNA viruses that infect epithelial cells of the skin and mucosa. HPV is the causal agent of cervix-uterine cancer and anogenital malignancies. However, HPV has been detected in extragenital tumors such as oral, esophageal, tonsillar, laryngea and lungs [1]. The HPV types that infect the genital mucosa can be divided into two groups. The high-risk types, including HPV16, 18, 31 and others, are frequently found in cervical cancers. By contrast, the low-risk types, including HPV6 and 11, also infect the genital epithelia, but are rarely detected in malignancies [28]

Considering the controversial reports on the association of HPV in breast carcinomas, the aim of this study was to explore HPV presence in this neoplasia among a female Argentinean population. Also to examine HPV genotype, particularly those of high risk and correlate their presence with clinical characteristics. This study demonstrated for the first time in Argentina the presence of HPV in a proportion of the malignant breast tissues, with a prevalence of low risk HPV11, followed by high risk HPV16.

## Materials and Methods

### Patients

The study was conducted on 61 fresh tissue biopsies of breast carcinoma, collected without any preselection criteria from the pathological archives of the Pathology Service of M. Villegas de Martinez Hospital. Tumors, which were typed according to the American Joint Committee on Cancer, included: 43 invasive ductal carcinomas, 10 invasive lobular, 2 papillary, 2 tubular, 1 adenocystic, 1 mucinous, 1 aprocrine and 1 comedocarcinoma. Patients’ age ranged from 35 to 92 years (median age 57 years).

This study has the approval of the Institutional Review Board and the Ethics Board of both M. Villegas de Martinez Hospital and Ricardo Gutierrez Children Hospital and is also in accordance with the Helsinki Declaration of 1975. A written informed consent was obtained from every patient.

### Polymerase Chain Reaction (PCR) for HPV Detection and Sequencing

DNA was extracted from tumor fresh biopsies using QIAamp DNA Mini Kit (QIAGEN GmbH, Hilden, Germany) following manufacturer’s instructions.

The presence of HPV DNA sequences was verified by amplification with two sets of primers. A first round with degenerated primers MY09, 5′-CGTCCMARRGGAWACTGATC-3′ and MY11, 5′-GCMCAGGGWCATAAYAATGG -3′, amplifying 450 bp long fragment in highly conserved region in L1 gene; and a second round with consensus primers GP5+, 5′-TTTGTTACTGTGGTAGATACTAC- 3′ and GP6+, 5′-CTTATACTAAATGTCAAATAAAAA-3′ generate 140 to 150 bp fragment of the L1 region of the virus [29]. Both systems of primers detect a broad spectrum of oncogenic and non-oncogenic HPV types. HPV positive Hela cell line (ATCC® Number: CCL-2) was used as positive control in all PCR reactions.

In a separate reaction tube, a set of primers for the beta-globin gene, 5′-GAAGAGCCAAG GACAGGTAC-3′ and 5′ -CAACTTCATCCACGTTCACC-3′, were incubated with the template DNA. The presence of a 260- base pair (bp) amplification product served as a control to monitor the amplification ability of a single copy gene.

HPV PCR products were separated by electrophoresis in 2% agarose gel stained with ethidium bromide and purified with QIAEXII gel extraction kit (Qiagen GmbH) according to manufacturer’s instructions. These purified PCR products were directly sequenced using Big Dye Terminator v3.1 kit (Applied Biosystems, Foster City, CA) in an automated Genetic Analyzer 3130×l (Applied Biosystems). HPV sequences were compared with published ones of known HPV types. At least two independent sequencing reactions were performed with the inner primers to confirm each sequence.

### E6 and E7 Analysis by RT-PCR

Total RNA in fresh biopsies was extracted using Trizol according to manufacturer instructions. In order to confirm efficient extraction, the quality of RNA from each sample was assessed by RT-PCR amplification of the ubiquitously expressed phosphoglycerate kinase gene (PGK), which acted as a control to ensure that only RNA was amplified (247 pb fragment), along with the absence of contaminating DNA (additional 600 bp fragment). Good quality RNA samples were chosen and 2 ug were used for cDNA synthesis using Superscript II RT kit (Invitrogen Inc., California, USA) according to the manufacturer’s instructions. Amplification was performed with specific primers (kindly given by Dr Luis Jave Suárez) for E6 HPV16 fwd 5′ CAGAGCTGCAAACAACTATAC 3′, rev 5′ AGTGGC TTTTGACAGTTAATA C 3', E7 HPV16 fwd 5′ GACAAGCAGAACCGGACAG 3′, rev5′ ATTCCTAGTGTGCCCATTAACA 3′, E6 HPV18 fwd 5′ GCGACCCTACAA GCTACCTGA T 3′, rev 5′ GCACCGCAGGCACCTTATTA 3′, E7 HPV18 fwd 5′ TGTCACGAGCAATTAAGCGACT 3′, rev 5′ CACACAAAGGACAGGGTGTTCA 3′.

### Statistical Analysis

Statistical analysis was performed using GraphPad Prism 4 software (GraphPad Software, Inc., San Diego California USA). Fisher’s exact test or Chi square tests (χ2) were used for statistical analysis when appropriate.

## Results and Discussion

Patients’ clinical characteristics and HPV association are shown in Table 1. Sixteen out of 61 patients (26%) were HPV positive for MY09/MY11 and GP5+/GP6+ amplification. Our results confirm and broaden earlier reports that also described a similar proportion of positive cases, such as the 23% HPV association with invasive ductal carcinoma, reported by Heng B et al in Australia [9], the 21% described in Japan by Khan et al [7] or the 25.9% HPV positive cases in Iran [16]. In line with these, Li et al conducted a meta-analysis and revealed that 24.5% of the breast carcinoma cases were associated with HPV, of which 32.4% occurred in Asia and 12.9% in Europe [12]. Increased HPV incidence in breast cancer was observed in Australia, were both Glenn et al [17] and Antonsson et al [22] described HPV DNA prevalence in the breast cancer samples of 50%. Conversely, only a 6.5% HPV association was described in China [21].

**Table 1:** HPV status according to clinical parameters in breast carcinoma cases.

Breast carcinoma characteristics	N	HPV status		*p*
		*pos*	*%*	
*Tumor histology*				
Invasive ductal	43	12	28	0.2487
Invasive lobular	10	1	11	
Papillary	2	2	100	
Tubular	2	1	50	
Adenocystic	1	0	0	
Mucinous	1	0	0	
Apocrine	1	0	0	
Comedocarcinoma	1	0	0	
***Total***	*61*	*16*	*26*	
Steroid hormone receptors^*^				
Positive	32	10	31	0.6956
Negative	10	2	20	
*Axillary lymph node status^#^*				
Positive	14	4	29	1.0000
Negative	22	6	27	

In Latin American population, Cantu de León et al described a 29.4% of HPV association with Mexican breast carcinoma [11] and Aguayo et al, an 8.7% of HPV16 presence in Chile [14]. In contrast, others studies performed in our region, in Brazil [25] and Mexico [26], described no evidence of HPV association with breast carcinoma. The findings reported by our study also support the notion for a viral association with breast carcinoma. In fact, in Argentina our group has previously also described EBV presence in 31% breast carcinoma patients, with a particular latency profile, not already observed in EBV-associated tumors [30]. Interestingly, 1/61 patients of this analyzed series harboured both HPV and EBV, pointing out that both viruses might contribute to the neoplasic process.

In this report, clinical parameters such as tumor histology, axillary lymph node status or estrogen and progesterone receptors were not statistically associated with HPV presence (p>0.05 χ^2^ test), in accordance with previous reports [7], [11], [14], [15]. However, Kroupis et al described the only breast cancer series harbouring high-risk HPV DNA sequences related to clinical parameters; in which those HPV+ cases displayed less estrogen-receptor and were proliferative [31]. Even though in the present study HPV presence was not statistically significant among histological tumor types, it is noteworthy that both papillary breast carcinoma cases, a rare histologic type, were HPV positive.

At present, about 130 HPV types were identified by their sequence of the gene encoding the major capsid protein L1 isolated from HPV associated diseases. Moreover, they can be also classified into high- and low-risk types depending upon their oncogenic potential. This is shown most clearly in the genital tract, in which there is regular or sporadic infection with about 30–40 types. These can be divided into those predominately associated with benign anogenital warts or condylomata, low-risk HPV types 6, 11 and their relatives, and those associated with anogenital cancers and the precursor lesions (intraepithelial neoplasia), particularly of the cervix, HPV 16, 18, 31, 33, 35, 45, and minor types. The most important players of cervical cancers are HPV 16, found in 50–70% of cases, and HPV 18, found in 7–20% of cases [32]. When HPV typing was performed by sequence analysis in a subgroup of 9 cases of our series, 4 cases were characterized as HPV 11, 3 as HPV 16 and 1 as HPV13. Notably, 1 case showed a co-infection with HPV 11 together with HPV 87. The presence of HPV11, a low risk type, was very remarkable since mostly high risk HPV 16 and 18 types were reported in breast carcinoma [17], [21], [22]. In Iran, high-risk HPV genotypes in breast cancer patients were the predominant types, but other genotypes (HPV-6, HPV-11, HPV-15, HPV-23, and HPV-124) were also detected [16]. Only de Villiers et al, in a German series, demonstrated that the most prevalent type in both breast carcinomas and nipples from the same patient was HPV 11, followed by HPV 6 [3]. In Argentina, the prevalence of HPV16 in high grade intraepithelial cervical lesions samples is 48.5% and 16.9% for HPV18; instead, invasive cervical cancer shows a prevalence of 59.5% for HPV16 and 17.6% for HPV18 [33].

HPV types in Latin American breast carcinoma patients, displayed prevalence for high risk HPV. In fact, in Chile only the HPV-16 genotype was present in positive breast carcinomas without co-infections with other HPV genotypes [14], while in Mexico, 66.6% were positive for HPV 16, 20% for HPV 18, and 13.4% were positive for both. High risk HPV types also prevail in breast carcinoma from other population. Damin et al [15] reported 56% HPV 16 and 40% HPV 18. In line with this, Li et al [12], in a meta-analysis, found that the four most commonly identified HPV types, in order of decreased prevalence, were HPV33, 18, 16, and 35. In Japan, the most frequently detected HPV genotype was HPV 16 (92%), followed by HPV 6 (46%), HPV 18 (12%), and HPV 33 (4%) [7]. Kroupis et al [31] identified a total of 21 high-risk viruses: 67% HPV 16, 14% HPV 59, 10% HPV 58, 5% HPV 73 and 5% HPV 82.

HPV analysis performed so far in this report enables exclusively evaluation of the presence of viral DNA, but do not inform on the possible viral activity or infection productivity. Therefore, evaluation of E6 and E7 mRNA in high risk genotypes may represent a good marker for HPV involvement in malignant transformation. In fact, a strong association between high risk mRNA-HPV presence and risk of neoplastic progression in cervical lesions has been described [34], [35]. In consequence, E6 and E7 mRNA-HPV presence in high risk phenotypes was suggested as a tool for early identification of cervical lesions that would evolve toward neoplastic transformation. Furthermore, HPV transcriptional activity was also found in most oropharyngeal squamous cell carcinoma [36]. E6 and E7 mRNA from high risk HPV16 and HPV18 evaluated in 9 cases with quality RNA from our series by RT-PCR were negative, demonstrating no transcriptional activity of high risk HPV types, as previously observed in Italy [37].

Although the route of transmission for the virus has not been determined, women positive for both breast and cervical cancers were found to be infected with the same HPV type in both tumors [37]. Furthermore, women with squamous precancer and women with glandular precancer in the cervix had a significantly higher risk of malignant breast tumors than the general female population from Norway [18]. Unfortunately, in our series HPV data in cervix tissue were not available to give rise to this assumption.

### Conclusions

The present study demonstrated for the first time in Argentina the presence of HPV in a significant proportion of the malignant breast tissues analyzed. Presence of low risk types, together with the absence of transcriptional activity for high risk types suggest that HPV presence may have a biological significance in breast carcinogenesis as a contributing factor to tumor development, but not as the tumor driven force. It should be pointed out that these observations need additional studies to be substantiated, especially by monitoring future breast cancer incidence amongst women vaccinated against high risk HPV type. Meanwhile, the present results may represent an important issue in virus-associated cancer, which represent potential tools for the development of specific therapies leading for patients with breast cancer.
